# The Coordination on Mobile Pandemic Apps Best Practice and Solution Sharing (COMPASS) Framework: Holistic Approach to Pandemic mHealth Apps

**DOI:** 10.2196/63920

**Published:** 2026-03-30

**Authors:** Christian Elsner, Michael Winter, Peter Gocke, Rüdiger Pryss, Dagmar Krefting

**Affiliations:** 1 Center for Artificial Intelligence, University of Lübeck, Lübeck, Germany; 2 Institute of Clinical Epidemiology and Biometry, University of Würzburg, Würzburg, Germany; 3 Institute of Medical Data Science, University Hospital of Würzburg, Josef-Schneider-Straße 2, Würzburg, 97070, Germany, 49 93120146471; 4 Digital Transformation Staff Unit, Charité, Berlin, Germany; 5 Department for Medical Informatics, University Medical Center Göttingen, Göttingen, Germany; 6 Campus Institute Data Science, University of Göttingen, Göttingen, Germany

**Keywords:** patient-reported outcome, pandemic app, mobile health, Network University Medicine, Coordination on Mobile Pandemic Apps Best Practice and Solution Sharing, COMPASS, technical framework, medical device regulation, medical devices, smartphone app, application, development, user engagement, app effectiveness, questionnaire, use

## Abstract

The COVID-19 pandemic has highlighted the crucial role of smartphone apps in public health, but it has also revealed challenges in terms of user acceptance and trust, as well as the secure integration of medical data. To overcome these, the COMPASS initiative (Coordination on Mobile Pandemic Apps Best Practice and Solution Sharing)—part of the German Network University Medicine (NUM) program—developed a structured framework for the coordinated development and delivery of pandemic apps, with a focus on usability, accessibility, security, and scalability. By incorporating expertise from 9 university hospitals and external partners, COMPASS provided a modular approach to pandemic app development that balances technology, regulation, and public acceptance. The framework includes governance, best practices, compliance, research compatibility, interoperability, and a scalable technology platform. In addition, standardized app components and templates were created to support an effective pandemic response. Real-world validation was provided by study-specific apps such as the Mainz Gutenberg Study COVID-19 app (University Medical Center Mainz) and the SentiSurv app (University Medical Center Mainz), which generated nearly 1 million data points from over 25,000 participants. COMPASS successfully developed study-specific apps, improved core functionalities, and contributed to larger digital health projects such as the InnovationHub CAEHR. Beyond its immediate applications, COMPASS serves as a scalable blueprint for future mobile health solutions, with a focus on data protection, user trust, and open-source collaboration. By integrating important technological, ethical, and user-oriented considerations, it sets a new standard for digital health innovation and ensures sustainable and widely accepted pandemic preparedness.

## Introduction

### Overview

The COVID-19 pandemic underscored the crucial role of smartphones in disseminating health information, particularly during lockdowns and social distancing [1]. This period saw a surge in pandemic-specific mobile apps aimed at managing public health crises [2]. However, their success depended on widespread adoption and public trust, especially when handling sensitive medical data and personal risk assessments [3]. Developers faced the challenge of balancing usability, security, and rapid deployment, while also addressing critical concerns regarding data protection and user control.

To tackle these challenges, the COMPASS initiative (Coordination on Mobile Pandemic Apps Best Practice and Solution Sharing [4]) was established as part of Germany’s Network University Medicine (NUM) program. From the outset, COMPASS aimed to create a structured framework for developing, evaluating, and deploying pandemic apps, leveraging expertise in science, technology, and legislation. Its core objective was to ensure that pandemic apps were effective, interoperable, and research-ready, facilitating their rapid and optimal use in future health emergencies.

A key challenge was the absence of clear legal, regulatory, and technical guidelines for integrating app-collected data into COVID-19 research while ensuring compliance with privacy standards [5]. This gap created uncertainties in assessing the effectiveness of these apps and limited their utility for stakeholders such as researchers, clinicians, and public health authorities.

Through interdisciplinary collaboration between scientists, industry, and academic partners, COMPASS developed an open-source, modular technology platform that allows for the rapid adaptation and reuse of pandemic apps. The initiative provided best practice guidelines, standardized app components, and frameworks to ensure compliance, usability, and security. Its approach bridges the gap between technological innovation and real-world health care application, demonstrating how mobile health (mHealth) solutions can be effectively integrated into pandemic management.

This paper highlights the interplay of key components within the COMPASS framework, emphasizing its strategic approach to coordinating mHealth solutions. By integrating best practices in app development, governance, interoperability, and data security, COMPASS establishes a sustainable model for future health crises. Beyond Germany’s NUM program, its findings contribute to global efforts in advancing digital health strategies for pandemic preparedness.

### Background Information on COMPASS

The COMPASS approach integrated the expertise and experience of 9 university hospitals and external partners in the development and use of pandemic apps. It established a collaboratively coordinated and standardized platform to ensure the legally compliant and interoperable deployment of such apps. Table 1 provides an overview of all 9 participating institutions, including their commonly used abbreviations and the affiliations of the authors contributing to this paper.

**Table 1.** Participating institutions in the COMPASS^a^ initiative and their areas of expertise.

Institution name	Abbreviation	Expertise
Charité – Berlin University Medicine (Peter Gocke)	CHA	Mobile apps, telemedicine, medical informatics, and hospital information systems
University Medicine Göttingen (Dagmar Krefting)	UMG	Health information exchange, medical informatics, data management, ethics, and wearable electronic devices
University Medicine at the Johannes Gutenberg University in Mainz (Christian Elsner)	UMM	Health information systems, mobile apps, telemedicine, project management, and health planning
University Hospital Würzburg (Rüdiger Pryss and Michael Winter)	UKW	Mobile apps, psychology, ergonomics, telemedicine, and public health informatics
University Hospital Cologne	UKK	Health information exchange and questionnaires
University Hospital Muenster	UKM	Multilingualism and mobile apps
University Hospital Regensburg	UKR	Mobile apps, public health informatics, and data collection
University Hospital Ulm	UKU	Mobile apps, telemedicine, psychology, and ergonomics
University Hospital Erlangen	UKEr	Data collection and mobile apps

a COMPASS: Coordination on Mobile Pandemic Apps Best Practice and Solution Sharing.

Table 2 provides a summary of the COMPASS project, outlining its primary objectives, duration, key participants, development principles, focus areas, sustainability efforts, and accessibility. This overview highlights the core components of the initiative, offering a quick reference to its scope, structure, and strategic direction.

**Table 2.** Key features and overview of the COMPASS^a^ initiative.

Feature	Description
Project name	COMPASS (Coordination on Mobile Pandemic Apps Best Practice and Solution Sharing)
Objective	To develop sustainable app technology for managing pandemics through collaborative solutions and best practices.
Duration	September 2020 to December 2021
Participants	9 University Hospitals forming an interdisciplinary team, including scientists from various fields, alongside partners from science and industry
Development basis	Entire development based on open-source principles
Key focus	Ensuring research compatibility, interoperability, and adherence to relevant medical research and care standards internationally and in Germany
Sustainability efforts	Developing sustainability structure concepts alongside the main project goals
Accessibility	Publicly accessible through an online directory for widespread use
Contact information	NUM-COMPASS website: [4]; Email: compass@unimedizin-mainz.de

a COMPASS: Coordination on Mobile Pandemic Apps Best Practice and Solution Sharing.

### Status Quo Before and After COMPASS

Despite being developed 4 years ago, smartphone-based medical apps remain in high demand, largely due to the growing emphasis on patient-reported outcomes (PROs) [6], which COMPASS actively supports. Studies highlight significant discrepancies between health care professionals’ assessments and patients’ actual symptom experiences [7], emphasizing the importance of PROs. Their impact is now widely recognized, with Newsweek recently including PROs as a criterion in its global hospital rankings [6]. Notably, research, particularly in oncology, demonstrates that PROs can improve patient survival rates [8].

However, 4 years after COMPASS, similar initiatives remain scarce. Our literature review [9,10] confirms this gap, and a technology search for freely available frameworks similar to COMPASS yielded minimal results [10]. Several factors contribute to this limited adoption [11], including stringent regulatory requirements, high development costs, and the rapid evolution of smartphone technology [12]. Additionally, many apps fail to prioritize user needs, lack comprehensive guidelines, and struggle to integrate into existing health care workflows. The broader digital health sector faces challenges in integrating health apps into clinical practice. While many apps exist, most lack clinical validation and remain underused. Key barriers include education and awareness gaps, missing digital formularies, limited workflow integration, and unclear reimbursement models. The digital divide also persists, preventing some populations from accessing or effectively using these technologies. Furthermore, the impact of health apps on clinician workload and burnout remains insufficiently studied.

### Related Work

A substantial body of literature exists on COVID-19 health apps [13,14]. However, many studies overlook a significant number of available apps [15,16], and those that do include them often fail to address broader mHealth frameworks for pandemic management. There are several initiatives related to COMPASS [17,18], some closely aligned and others more general in scope. However, none provide the comprehensive, interdisciplinary approach that COMPASS offers. Research has consistently highlighted barriers to adoption [19,20], reinforcing the need for fundamental frameworks such as COMPASS in both mHealth apps and PRO integration.

## International Initiatives, Frameworks, and Projects Related to COMPASS

In this document [21], COMPASS identified and compared national and international initiatives, frameworks, and projects related to pandemic apps. Our overall goal was to evaluate how such initiatives align with the goals of the COMPASS project and provide insights for future developments in mHealth apps.

### Objectives

Our objectives were as follows:

1. Identify international activities related to pandemic apps beyond Germany.
2. Analyze useful insights from these initiatives for future pandemic preparedness.
3. Assess international frameworks that focus on mHealth apps.
4. Compare findings with COMPASS to determine gaps, best practices, and interoperability.

### Instruments

The following instruments were used:

1. Contacting international organizations with expertise in mHealth apps.
2. Conducting an online search for pandemic-related projects and frameworks.
3. Surveying international partners in the COMPASS network.

The findings showed that no direct equivalent to COMPASS exists internationally at the time of development, highlighting the necessity of a structured, interoperable pandemic app framework. However, several international initiatives and frameworks provide valuable insights and technical solutions (Table 3).

**Table 3.** Relevant international initiatives.

International initiative	Description
GNSS (European Global Navigation Satellite Systems Agency)	Provides navigation and positioning data crucial for GPS-based pandemic apps, ensuring the accuracy, integrity, and availability of location-based services [22].
UN-DESA^a^ Digital Government Initiatives (COVID-19 Response)	Compendium of global digital government responses to COVID-19, helping countries share best practices [23].
WHO^b^ Academy and WHO Info Apps	Mobile tools providing COVID-19 guidance, virtual training, and educational resources for health care professionals [24].

a UN-DESA: United Nations Department of Economic and Social Affairs.

b WHO: World Health Organization.

In addition, we identified 14 pandemic-related frameworks, 13 of which focus on contact tracing (Table 4). The most notable ones are described below.

**Table 4.** Pandemic-related frameworks.

Framework	Description	Usage
ViraTrace [25]	Uses advanced proximity detection models to improve contact tracing. Integrated into India’s Aarogya Setu app (National Health Authority, India).	Aarogya Setu (India)
PEPP-PT (Pan-European Privacy-Preserving Proximity Tracing) [26]	Aimed at standardizing contact tracing in Europe while ensuring GDPR^a^ compliance.	ROBERT (France) and StopCovid (Georgia)
Coalition Network [27]	Bluetooth-based contact tracing.	Coalition App (Global)
Google-Apple Exposure Notifications System [28]	A global contact tracing protocol adopted by several national apps.	Corona-Warn-App (Germany), COVID Alert (Canada), Immuni (Italy), etc
DP-3T (Decentralized Privacy-Preserving Proximity Tracing) [29]	Open-source protocol ensuring privacy-preserving exposure notifications.	Coronalert (Belgium)
PACT (MIT’s Private Automated Contact Tracing) [30]	Privacy-first contact tracing protocol developed with academic institutions and hospitals.	COVIDSafe (United States)
SORMAS (Surveillance Outbreak Response Management and Analysis System) [31]	Monitors and analyzes infection outbreaks in Germany.	Used in German health departments.

a GDPR: General Data Protection Regulation.

### Survey Results

The international survey assessed pandemic app initiatives and COMPASS’s relevance. Key insights:

1. Respondents confirmed that more international collaboration is needed.
2. The open-source approach of COMPASS was widely supported.
3. A few pandemic apps were identified, such as OstaniZdrav (Republic of Slovenia) [32] and Virusafe (Council of Ministers of the Republic of Bulgaria) [33].
4. There was no clear consensus on additional missing focal points in COMPASS.
5. Discussions with the European Health Futures Forum helped increase international visibility.

## Strategic Aims and Objectives of the COMPASS Initiative

**Figure 1.**
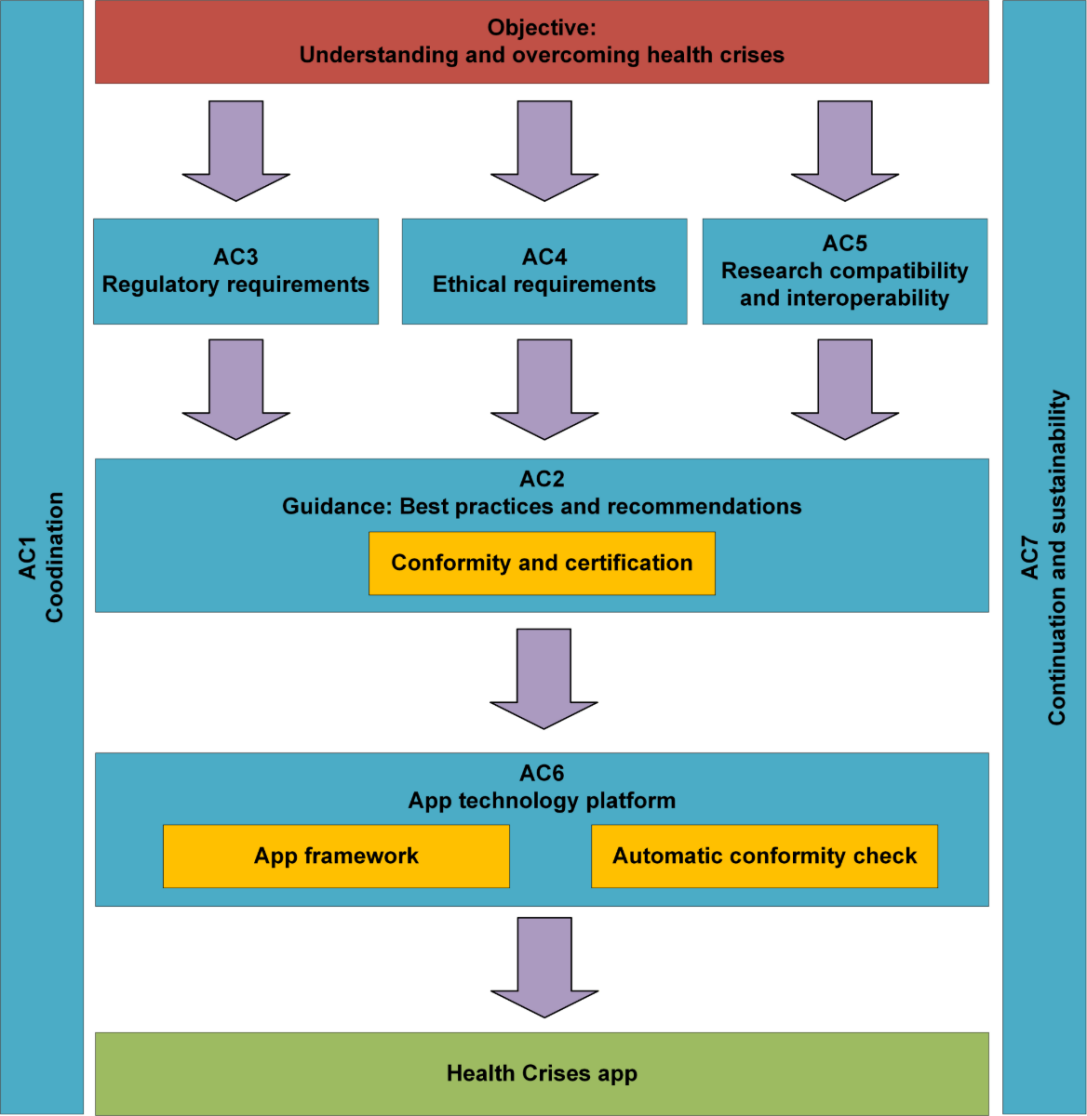
Organizational structure and workflow of the Coordination on Mobile Pandemic Apps Best Practice and Solution Sharing initiative’s activity clusters.

COMPASS was structured into 7 activity clusters (ACs), as illustrated in Figure 1. Table 5 provides a concise summary of the key tasks and outcomes across these clusters.

**Table 5.** Number of participants and data records for studies using COMPASS^a^ apps.

Study	Number of participants (n) and data records (D)
Mainz Gutenberg Study COVID-19 app [34]	n=7500; D=227,000
SentiSurv [35]	n=19,700; D=654,000
SAM^b^ [36]	n=110; D=568
FORTEe [37]	n=12; D=not available

a COMPASS: Coordination on Mobile Pandemic Apps Best Practice and Solution Sharing.

b SAM: Self-Monitoring App for Employee Health.

### AC1: Coordination

A governance structure was established to monitor project milestones, enable rapid response to deviations, and ensure alignment with AC2. Effective communication and coordination with international pandemic networks were prioritized to synchronize technical developments.

### AC2: Guidance (Best Practices and Recommendations)

Best practices and guidelines were developed to enhance the effectiveness, efficiency, and research compatibility of pandemic apps. A digital knowledge database was created to support partners in meeting regulatory requirements. Advisory structures were also established to disseminate these guidelines.

### AC3: Regulatory Requirements

Regulatory demands for pandemic apps proved more stringent than for standard medical apps, requiring a different development approach. Guidelines were formulated to address these challenges and ensure compliance with fast-evolving requirements.

### AC4: Ethical Requirements

Ethical considerations were critical due to public concerns over mandatory app usage during pandemics. The cluster focused on trust-building measures to help citizens and researchers navigate the ethical implications of pandemic apps.

### AC5: Research Compatibility and Interoperability

Key requirements for using app-collected data in pandemic research were identified. Concepts and recommendations were developed to guide app configuration, which were integrated into AC2. A generic HL7 FHIR (Fast Healthcare Interoperability Resources) interface was initially created and iteratively expanded to accommodate specific data models [38].

### AC6: App Technology Platform

A modular technology platform was developed, featuring templates, technical components, and a semiautomated compliance check for regulatory adherence. Reference implementations for both native and cross-platform development were provided, all under open-source licenses, ensuring quick adaptability for future pandemics.

### AC7: Continuation and Sustainability

Long-term strategies were designed to ensure the ongoing operation of the platform, including regular updates aligned with legal and scientific advancements. A certification framework was proposed, alongside a responsive advisory structure for future pandemics. Maintenance efforts focused on keeping apps compatible with evolving mobile operating systems.

## Overview of Solutions Orchestrated Through COMPASS

Upon its successful completion, the COMPASS project achieved significant milestones in 3 key areas: the development and implementation of study-specific apps, the enhancement of technical components, and the application of nontechnical insights for future use.

### Pillar 1: Development and Implementation of Study-Specific Apps

This pillar highlights the successful deployment of multiple study-related apps (Table 5):

1. Mainz Gutenberg Study COVID-19 app: used for monitoring COVID-19 within the scope of the Mainz Gutenberg Study.
2. DZHK App for Medication and Anamnesis (Universitätsmedizin Göttingen): employed at the German Center for Cardiovascular Research for recording medication and medical histories.
3. SentiSurv app: applied in Mainz for the continuous monitoring of the SARS-CoV-2 pandemic.
4. SAM (Self-Monitoring App for Employee Health; Universitätsmedizin Mainz): developed under the NUM project egePan Unimed, this app is currently under evaluation and further development in the NUM subproject “PREPARED” of the second funding phase.
5. Stress Radar (Universität Würzburg): designed to assess the psychological stress of employees.
6. FORTEe Study (Universitätsmedizin Mainz): conducted in the realm of pediatric oncology, in collaboration with IZKS, as part of the FORTEe project. This involved the use of both web and native apps.

### Pillar 2: Advancement of Technical Components

This domain primarily focused on the extensive development of the questionnaire editor, with detailed technical specifications available at [39]. Simultaneously, ongoing maintenance and enhancement efforts have ensured the optimization of key components, as outlined in [40].

### Pillar 3: Continuation and Use of Nontechnical Findings

For example, within the InnovationHub CAEHR project, findings on Medical Device Regulation and eHealth insights have been successfully integrated and reused. Regular high-frequency monthly inquiries about these results ensure their continued relevance and refinement.

## Structured Summary of COMPASS Results

Table 6 below provides a structured summary of COMPASS results across the ACs.

**Table 6.** Summary of tasks and work results across COMPASS^a^ initiative activity clusters.

Activity cluster	Task	Work results
AC1	Synchronize and document the work content of national and international groups	Identified relevant projects (4), frameworks (12), and artifacts (9) that were developed internationally during COVID-19.
AC2	Conformity check for pandemic health apps Prototype advisory structures to attract further institutions Job description for consulting and inquiry management Prototype advisory structures to attract further university hospitals	Developed modules for evaluating the conformity of health apps with established protocols, focusing on privacy and technical specifications. Developed a scalable framework to facilitate collaboration and support among various stakeholders. Defined a job role focused on consulting and managing inquiries, enhancing the use of COMPASS components in health care. Created advisory structures for interacting with university hospitals, focusing on guidelines and services synchronization.
AC3	Explanation of the technical development variants for pandemic apps Interim results best practices and checklists regulatory requirements Regulatory field report Best practices and checklists	Analyzed and compared different development approaches for pandemic apps, focusing on performance, costs, and regulatory compliance. Outlined preliminary checklists for developing regulation-compliant pandemic apps. Examined regulatory frameworks to ensure that pandemic app development processes comply with standards. Provided guidance on regulatory compliance for mobile health apps, especially under pandemic conditions.
AC4	Catalog of best practices and checklists usability, diversity, and licensing Study design and recruitment strategy for the social acceptance study Literature review on the evidence of pandemic apps Literature review on ethical recommendations and normative governance guidelines Best practices and guidelines of ethical-social requirements	Analyzed usability, diversity, and licensing issues, producing best practices and checklists. Designed and executed studies to understand public attitudes toward pandemic apps and their effectiveness. Assessed the effectiveness of pandemic apps through scientific, user, and legal lenses, using bibliometric analyses. Reviewed ethical and governance guidelines for pandemic app development, focusing on privacy, transparency, and participation. Explored usability, diversity, and licensing in pandemic apps, providing recommendations for improvement based on user feedback.
C5	Research compatibility and interoperability FAIR^b^ principles Specification of data and metadata models Concept for ensuring research compatibility for apps Late mapping scheme	Identified requirements and recommendations for the use of app-collected data in pandemic research. Focused on implementing FAIR principles to improve the management and use of research data. Developed specifications for data and metadata models to ensure compatibility and integration with the NUM^c^ platform. Provided concepts and recommendations for integrating app-collected data into pandemic research, ensuring interoperability and scientific reuse. Addressed compatibility issues for data collected prior to coordination with the NUM COMPASS project.
AC6	App framework Templates: privacy statements, terms of use, and further information	Developed a platform containing frameworks for essential components of pandemic apps. Compiled templates for legal and privacy aspects necessary for pandemic apps.
AC7	Survey of existing guidance structures and economic analysis of guidance operation Survey of technology platforms	Developed strategies for sustainable management of the COMPASS platform, incorporating best practices and regular updates. Explored strategies for sustainable operation and continuity of technology platforms used in pandemic management.

a COMPASS: Coordination on Mobile Pandemic Apps Best Practice and Solution Sharing.

b FAIR: Findable, Accessible, Interoperable, Reusable.

c NUM: Network University Medicine.

## Scientific Output of COMPASS

The COMPASS project has shared its findings through numerous scientific publications (Table 7), with 13 papers covering the development, implementation, and evaluation of COVID-19 apps within the NUM-COMPASS framework. Key contributions include the integration of PROs into the centralized CODEX research platform (Network University Medicine), analyses of regulatory and usability aspects, and the promotion of interoperable standards using the GECCO (German Corona Consensus) dataset. Additionally, studies examined public perception and acceptance, fostered community-driven open-source solutions, and developed educational materials to support data standardization and interoperability. These efforts strengthen data sharing and research capabilities during and beyond the pandemic.

**Table 7.** Overview of scientific publications resulting from the COMPASS^a^ initiative.

Title	Description	Reference
Medical Device Regulation Efforts for mHealth Apps During the COVID-19 Pandemic—An Experience Report of Corona Check and Corona Health	This paper uses specific examples to analyze the costs associated with rapidly implementing COVID-19 apps in compliance with the Medical Devices Act.	[41]
Integration of Patient-Reported Outcome Data Collected Via Web Apps and Mobile Apps into a Nation-Wide COVID-19 Research Platform Using Fast Healthcare Interoperability Resources: Development Study	This paper describes the integration of data collected via the COMPASS reference app framework into the central German CODEX platform to facilitate secondary research. The paper demonstrates how patient-reported outcome data can be standardized, shared, and reused across various research studies to enhance COVID-19 research.	[38]
Motivating Developers to Use Interoperable Standards for Data in Pandemic Health Apps	This work discusses COMPASS in general.	[42]
Public Perception of the German COVID-19 Contact-Tracing App Corona-Warn-App	This article examines the public perception (based on the store reviews) of the official German Corona Warn app to derive lessons for future pandemics.	[43]
Relevant Aspects for Sustainable Open-Source Pandemic Apps and Platform Deployment with Focus on Community Building	Based on a literature review, a classification for the long-term success of open-source software was developed. This paper introduces a classification centered on five distinct categories: (1) structural decisions, (2) revenue generation, (3) user focus, (4) openness, and (5) community building. Developed within the NUM-COMPASS project, the classification focuses on pandemic apps and the open-source framework. We offer insights into the community-building dimension by discussing the factors essential for creating sustainable communities.	[44]
Pandemic Apps: Broad Acceptance	A representative telephone survey on pandemic apps for research in Germany reveals that many Germans are willing to share their health data with researchers. This paper presents the results of the survey.	[45]
Making COVID-19 data interoperable: the COMPASS GECCO recommendations	We analyzed data standardization in pandemic apps and developed training materials for researchers and developers with limited knowledge of semantic and syntactic interoperability. This includes a how-to guide and a conformity checkpoint for data models, using GECCO^b^ as the reference. We identified weaknesses in interoperable data collection and interviewed developers from other COVID-19 projects to understand their needs. An executive summary was created to raise awareness among executives about NUM-COMPASS. Additionally, a First Contact Package was devised to inform researchers and developers. It simplifies the concept of interoperable data capturing and guides them through the FAIR^c^ principles and the GECCO Implementation Guide.	[46]
Tutorial: Configure the NUM-Compass App for your interoperable NUM study (in German)	A full day course with hands-on configuration of the COMPASS backend and the web app on a specific use case in configured virtual machines for each participant.	[47]
Exploring the Usability of the German COVID-19 Contact Tracing App in a Combined Eye Tracking and Retrospective Think Aloud Study	This article examines the usability of the official German Corona Warn app to derive lessons for future pandemics.	[48]
Corona Health— A Study- and Sensor-based Mobile App Platform Exploring Aspects of the COVID-19 Pandemic	A study was conducted within an existing COVID-19 app to assess users’ knowledge of regulations, as part of the COMPASS project.	[49]
Towards FAIR Patient Reported Outcome: Application of the Interoperability Principle for Mobile Pandemic Apps	During the COVID-19 pandemic, numerous apps were developed to collect and track medical data. Despite addressing similar aspects, diverse data models and formats prevent joint analysis of the data. The NUM-COMPASS project, part of the German COVID-19 Research NUM^d^, created a coordination and technology platform for researchers and app developers. This platform ensures data collection complies with the GECCO, focusing on interoperability as part of the FAIR principles. This paper details how NUM-COMPASS implements these principles to support joint analysis of data from various app-based studies.	[50]
Attitudes Towards Mobile Apps for Pandemic Research Among Smartphone Users: Results from a National Survey in Germany	The aim of this study was to explore the potential for the acceptance of research-oriented apps in the German population. To this end, we identified distinctive attitudes toward pandemic apps and data sharing for research purposes among smartphone users in general and with a focus on differences in attitudes between app users and nonusers in particular.	[51]
Toward an interoperable Ecosystem within the Network University Medicine – adopting the Compass App to the central CODEX platform	Within the NUM, different projects have been carried out to support the understanding and handling of the COVID-19 pandemic. The CODEX project has developed a platform to collect and provide clinical routine data of COVID-19 patients in the GECCO format, but also to provide a platform for app-based study data. On the other hand, the COMPASS project has focused on an open-source framework for research-compatible pandemic apps. In this work, we adopted the Compass app to connect directly to the central CODEX platform.	[52]

a COMPASS: Coordination on Mobile Pandemic Apps Best Practice and Solution Sharing.

b GECCO: German Corona Consensus Dataset.

c FAIR: Findable, Accessible, Interoperable, Reusable.

d NUM: Network of University Medicine.

## Narrative Summary

The following section provides a narrative summary of key findings from COMPASS. These findings are currently accessible in various German-language reports on the COMPASS website. A complete list of these documents is referenced in Table 8, and they have also been archived for long-term access in the following GitHub repository. We recommend referring to the files in this repository, which can be easily translated using tools such as ChatGPT (OpenAI).

**Table 8.** Documents produced by the COMPASS^a^ project.^b^

Document no.	Description
Document 1	Documentation and comparison of work content from national and international groups (German)
Document 2	Conformity check for pandemic health apps
Document 3	Prototype of advisory structures for the acquisition of additional university hospitals
Document 4	Job description for consulting and request management
Document 5	Prototype of advisory structures for the acquisition of additional university hospitals
Document 6	Explanation of technical development options for pandemic apps
Document 7	Regulatory experience report
Document 8	Regulatory best practices and checklists
Document 9	Catalog of best practices and checklists on usability, diversity, and licenses
Document 10	Study design and recruitment strategy for the societal acceptance study
Document 11	Literature review on the evidence of pandemic apps
Document 12	Literature review on ethical recommendations and normative governance guidelines for the development, design, and application of pandemic apps
Document 13	Best practices and guidelines for ethical and societal requirements
Document 14	Research compatibility and interoperability
Document 15	FAIR^c^ principles
Document 16	Specification of data and metadata models: Part 1
Document 17	Specification of data and metadata models: Part 2
Document 18	Concept for ensuring research compatibility in apps
Document 19	Late mapping scheme
Document 20	Technical app framework
Document 21	Templates: Privacy policies, terms of use, and additional information
Document 22	Survey of existing guidance structures and economic analysis of guidance operations
Document 23	Survey of technology platforms and analysis of technology operations

a COMPASS: Coordination on Mobile Pandemic Apps Best Practice and Solution Sharing.

b Documents can be accessed in the following repository: [53]. Document 20 can be accessed here: [40].

c FAIR: Findable, Accessible, Interoperable, Reusable.

During discussions with researchers throughout the pandemic and shortly thereafter, several critical insights emerged regarding COMPASS and its applications in pandemic-related apps specifically, as well as mHealth apps in general. A key takeaway is that a technical solution must be available both as a web app and as a native app in app stores to provide maximum flexibility for different user needs and scenarios. COMPASS successfully implemented this approach within a short timeframe, and the corresponding codebase can be found at [54].

Furthermore, it became evident that many mHealth apps require integrated questionnaire functionalities. The ability to configure questionnaires within a web-based tool—ideally with access to preexisting standardized questionnaires—is essential. Additionally, adherence to standards such as FHIR should be ensured, and all questionnaire elements should be fully annotated, not only at a technical level (eg, sliders and yes or no questions) but also semantically. To address this, a dedicated questionnaire editor was developed. Originally, the project aimed to integrate this tool with the Medical Data Models (MDM) portal of the University of Münster. However, due to time constraints, this integration could not be completed within the project’s timeframe. The MDM portal [55] provides extensive access to standardized medical data models.

Another significant achievement of COMPASS was the development of a technical framework for the automated testing of mHealth apps (Table 8).

As shown in Table 8, additional findings were obtained on various other aspects. Future developments will determine the extent to which these findings will play a role in further research and application.

## Critical Discourse and Evaluation of COMPASS

The COMPASS project invites critical discourse on its approach and outcomes, particularly its innovative use of smartphone-based health apps—both a strength and a point of contention.

A key concern is the balance between technological advancement and user privacy. While COMPASS prioritizes ethical data collection and user consent, the extensive collection of health-related data raises questions about privacy safeguards and potential security risks. Given the sensitivity of medical data, ensuring robust protections against breaches remains a crucial challenge.

Another area of scrutiny is public trust and app adoption. While COMPASS aims to improve user confidence and uptake, its effectiveness in overcoming skepticism—especially amid misinformation and concerns surrounding health technologies—remains an open question. Addressing diverse public perceptions is essential for broader acceptance.

The project’s open-source framework enhances accessibility and adaptability; yet, it also poses challenges for standardization and interoperability. Many health care infrastructures lack seamless integration capabilities, making it critical to ensure that pandemic apps remain both interoperable and functionally robust.

While COMPASS’s interdisciplinary approach is a strength, it also introduces coordination challenges. Effective communication and integration across diverse expertise and disciplines are essential but logistically complex.

Finally, the sustainability and scalability of COMPASS require continued evaluation. Although the project established a foundation for long-term collaboration, its ability to adapt to evolving health care challenges remains to be tested.

Despite these challenges, COMPASS represents a significant step forward in health technology and pandemic management. Addressing privacy concerns, public trust, standardization, interdisciplinary collaboration, and long-term sustainability will be key to refining future health technology initiatives and ensuring their lasting impact.

## Discussion: Implications and Future Considerations

### Overview

The COMPASS initiative demonstrates that effective pandemic mHealth apps require systemic coordination beyond technological innovation. While designed for pandemic response, its framework addresses fundamental challenges in health care digitalization: balancing rapid innovation with regulatory compliance, integrating patient-generated data into research infrastructure, and ensuring interoperability across fragmented systems. The emphasis on PROs and standardized data collection has implications extending to chronic disease management, remote monitoring, and routine clinical care. However, COMPASS’s experience raises critical questions about whether such frameworks can be sustained outside crisis contexts that create urgency and overcome traditional implementation barriers.

Despite COMPASS’s open-source design and adherence to international standards (HL7 FHIR and FAIR [Findable, Accessible, Interoperable, Reusable] principles), its transferability across different contexts remains uncertain. The absence of similar international initiatives suggests deeper structural barriers: fragmented health care systems, varying regulatory environments, and insufficient incentives for collaborative development in health care. Moreover, the framework’s applicability to resource-limited settings or endemic disease management requires further exploration.

User adoption challenges persist beyond technical solutions. Digital health apps inherently exclude populations without devices or digital literacy, potentially exacerbating health disparities during crises. While COMPASS surveys suggest German citizens’ willingness to share health data, this may not generalize globally, and trust deficits reflect broader concerns about data capitalism and surveillance that technical measures like encryption cannot fully address. Future frameworks must explicitly engage with health equity and meaningful public participation in governance.

A notable gap in COMPASS evaluation is a detailed analysis of integration into clinical workflows and impact on health care provider workload. The paper acknowledges that clinician burden remains insufficiently studied, yet this represents a critical implementation challenge for already strained health care systems. While COMPASS’s modular design addresses adaptability, questions remain about long-term maintenance responsibilities, evolution alongside changing technology, and the effectiveness of proposed sustainability structures beyond initial project funding.

Several critical research questions emerge: how do COMPASS apps perform against alternatives in terms of data quality and public health impact? What organizational factors enable successful adoption? How can apps serve diverse populations with varying digital access? What business models support ongoing maintenance beyond project funding? The nearly 1 million data points collected demonstrate feasibility but not effectiveness or translational impact.

COMPASS’s navigation of Medical Device Regulation offers valuable lessons for regulatory frameworks during health emergencies, suggesting the need for expedited pathways that maintain safety standards. The international fragmentation in pandemic app approaches indicates potential value in World Health Organization (WHO)–convened coordination mechanisms, though questions of sovereignty and resource distribution complicate such efforts.

The evaluation focuses on process and outputs rather than health outcomes. Whether COMPASS apps improved pandemic management or public health decision-making remains unclear. The paper predominantly reflects developer and researcher perspectives rather than end users, health care providers, or public health officials—stakeholder voices that should be centered in future work.

The 4-year gap since completion offers an opportunity to assess actual sustainability: which components remain actively maintained? Did proposed advisory structures and certification frameworks materialize? What barriers emerged? These questions are critical for understanding whether COMPASS achieved its objectives and informing future initiatives.

In summary, COMPASS demonstrates that coordinated, interdisciplinary pandemic app development is feasible, but translating technical capabilities into sustained public health impact requires addressing deeper challenges of trust, equity, clinical integration, and institutional sustainability. The true test lies not in what COMPASS achieved during its funding period, but whether its components influence subsequent digital health initiatives and improve preparation for future pandemics. COMPASS should be understood as the beginning of a longer conversation about digital health infrastructure rather than its conclusion.

### COMPASS: A Comprehensive Summary

COMPASS is a cohesive initiative in medical informatics, bringing together interdisciplinary expertise to develop a structured platform for coordinating pandemic apps and technology. It underscores the role of smartphones in health communication and the importance of public trust and adoption for effective pandemic response. By integrating technology into health care, COMPASS provides a practical framework for future medical research and public health strategies, particularly in health crises. Through its collaborative approach, it addresses current challenges while laying the groundwork for future advancements in health care technology and research.

## Abbreviations

AC: activity cluster
COMPASS: Coordination on Mobile Pandemic Apps Best Practice and Solution Sharing
FAIR: Findable, Accessible, Interoperable, Reusable
FHIR: Fast Healthcare Interoperability Resources
GECCO: German Corona Consensus Dataset
MDM: Medical Data Models
mHealth: mobile health
NUM: Network University Medicine
PRO: patient-reported outcome
SAM: Self-Monitoring App for Employee Health
WHO: World Health Organization
